# Advancing Optical Imaging for Breast Margin Assessment: An Analysis of Excisional Time, Cautery, and Patent Blue Dye on Underlying Sources of Contrast

**DOI:** 10.1371/journal.pone.0051418

**Published:** 2012-12-10

**Authors:** Torre M. Bydlon, William T. Barry, Stephanie A. Kennedy, J. Quincy Brown, Jennifer E. Gallagher, Lee G. Wilke, Joseph Geradts, Nimmi Ramanujam

**Affiliations:** 1 Department of Biomedical Engineering, Duke University, Durham, North Carolina, United States of America; 2 Department of Biostatistics and Computational Biology, Dana-Farber Cancer Institute, Boston, Massachusetts, United States of America; 3 Zenalux, Research Triangle Park, North Carolina, United States of America; 4 Department of Surgery, Duke University Medical Center, Durham, North Carolina, United States of America; 5 Department of Surgery, University of Wisconsin School of Medicine and Public Health, Madison, Wisconsin, United States of America; 6 Department of Pathology, Duke University Medical Center, Durham, North Carolina, United States of America; University Medical Centre Utrecht, The Netherlands

## Abstract

Breast conserving surgery (BCS) is a recommended treatment for breast cancer patients where the goal is to remove the tumor and a surrounding rim of normal tissue. Unfortunately, a high percentage of patients return for additional surgeries to remove all of the cancer. Post-operative pathology is the gold standard for evaluating BCS margins but is limited due to the amount of tissue that can be sampled. Frozen section analysis and touch-preparation cytology have been proposed to address the surgical needs but also have sampling limitations. These issues represent an unmet clinical need for guidance in resecting malignant tissue intra-operatively and for pathological sampling. We have developed a quantitative spectral imaging device to examine margins intra-operatively. The context in which this technology is applied (intra-operative or post-operative setting) is influenced by time after excision and surgical factors including cautery and the presence of patent blue dye (specifically Lymphazurin™, used for sentinel lymph node mapping). Optical endpoints of hemoglobin ([THb]), fat ([β-carotene]), and fibroglandular content via light scattering (<µ_s_’>) measurements were quantified from diffuse reflectance spectra of lumpectomy and mastectomy specimens using a Monte Carlo model. A linear longitudinal mixed-effects model was used to fit the optical endpoints for the cautery and kinetics studies. Monte Carlo simulations and tissue mimicking phantoms were used for the patent blue dye experiments. [THb], [β-carotene], and <µ_s_’> were affected by <3.3% error with <80 µM of patent blue dye. The percent change in [β-carotene], <µ_s_’>, and [β-carotene]/<µ_s_’> was <14% in 30 minutes, while percent change in [THb] was >40%. [β-carotene] and [β-carotene]/<µ_s_’> were the only parameters not affected by cautery. This work demonstrates the importance of understanding the post-excision kinetics of *ex-vivo* tissue and the presence of cautery and patent blue dye for breast tumor margin assessment, to accurately interpret data and exploit underling sources of contrast.

## Introduction

Breast conserving surgery (BCS) is a recommended treatment for early-stage breast cancer and for breast cancers that have been reduced in size by neoadjuvant therapy. The goal of BCS is to excise the tumor along with a margin of normal tissue, while preserving as much of the normal breast tissue as possible. Unfortunately, as many as 18–72% of patients undergoing BCS require repeat surgeries due to a close or positive surgical margin diagnosed post-operatively and thus, require a re-excision surgery to achieve cancer free margins [1]–[9]. These re-excision surgeries are not only a burden to patients financially but also physically and psychologically and can delay recommended adjuvant therapies. Additionally, 10–36% of women requiring re-excision will undergo mastectomy which significantly alters a patient’s initial treatment decision [10]. The large variation in re-excisions is thought to be due to differences in surgeon’s training, in the definition of a close margin, and in the perceived risk of focally positive margins versus extensive involvement [10].

Histopathology is the current gold standard for determining surgical margin status. At many hospitals, including Duke University Medical Center (DUMC), the standard of care is to grossly section the specimen into 3 mm slices perpendicular to the long axis of the specimen. The tissue slices are then further sectioned and from each of the resulting paraffin blocks, a 5 µm thick section is taken for staining and histological review. The pathologic margin status is an important predictor of local recurrence of an invasive or *in situ* cancer after BCS [11], [12]. Thus, re-excision of the tumor margin is essential to reduce the risk of local recurrence [13]. In post-operative pathology, it is not feasible to section and analyze the entire specimen, especially when the specimens are large. This issue was evaluated by Guidi et al. [14] where they looked at the presence of tumor in perpendicularly sliced sections of the inked margin (the type of analysis done here at DUMC) versus evaluating tissue from an *en face* cut of the margin (i.e. a shaved margin). They found that of 69 positive shaved margins, only 42 inked margins were found to be positive, indicating that residual carcinomas may be missed with the current approach of sampling tissue every couple of millimeters.

A small number (less than 5%) of hospitals that perform BCS currently utilize intra-operative cytologic or pathologic analysis of tumor margins. Touch-preparation (touch-prep) cytology is a technique in which cells on the surface of the tissue are transferred to glass slides by touching the specimen to the glass, and are then stained for pathologic observation. For frozen section analysis, the tissue is frozen and select microscopically thin sections are cut from the specimen for pathologic observation. Typically a much smaller fraction of the tumor margin is sampled in frozen section than in post-operative pathology. Touch-prep cytology and frozen-section analysis can reduce surgical re-excision rates; reported sensitivities and specificities for touch-prep are 38–100% and 83–100%, respectively [15]–[22]. Sensitivity of frozen section ranges from 59–91% and specificity ranges from 86–100% [18], [23]–[29]. Although these two approaches have been shown to be beneficial to the surgeon, there are a number of limitations with each. Both procedures are time consuming and require special expertise by a pathologist at the time of surgery. Additionally, touch-prep cytology allows for the evaluation of the whole lumpectomy surface but is not capable of detecting close margins since only cells at the specimen surface are sampled. Frozen section analysis may not be utilized on every patient but may be determined in collaboration with the surgeon, pathologist, and radiologist after a laborious process of gross examination and specimen mammography [29]. Sampling issues are also a problem since the entire specimen cannot be evaluated.

The above discussion points to the fact that surgery to remove the cancer and obtain clear margins is a collaborative effort between the surgeon and the pathologist (and in some institutions, the radiologist). In spite of this, there can be substantial variability in the prediction of positive margins in the intra-operative and post-operative settings. Surgeons do not have adequate intra-operative assessment tools to ensure that the cancer has been completely removed at the time of first surgery. Pathologists do not have adequate tools for sampling from areas on large tumor margins. The lack of these capabilities represents a significant unmet clinical need for margin assessment for both the surgeon and pathologist.

Optical imaging of tissue is an attractive solution to this problem because it is relatively fast and non-destructive. Optical techniques can also measure features related to the histological landscape without the need for labels. Table 1 provides a breakdown of the different optical tools that have been leveraged to measure breast tissue constituents for different applications in breast cancer ranging from diagnostic biopsy to margin assessment to monitoring of response to neoadjuvant therapy. This table shows that no matter what tool is used, the primary sources of contrast in breast tissue are scattering (which primarily reflects the fibroglandular content of breast tissue), lipid and carotenoid concentration (which reflects the fatty content of the breast tissue content), hemoglobin (which reflects tissue vascularity), and in the case of fluorescence, metabolism of the tumor cells.

**Table 1:** Optical sources of contrast in breast tissue.

Source of Contrast	DRS and ESS[37,41,43,48–50,55–59]	NIR spectral imaging[60]–[64]	FL spectroscopy[37], [49], [50], [55], [58], [65]	Raman spectroscopy[32], [66]–[68]	OCT[33], [69], [70]
Oxy-hemoglobin	✓	✓			
Deoxy-hemoglobin	✓	✓			
Heme				✓	
β-carotene or carotenoids	✓			✓	
Lipids		✓		✓	
Water		✓			
Scattering	✓	✓			✓
Collagen			✓		
NADH			✓		
FAD			✓		

DRS = diffuse reflectance spectroscopy; ESS = elastic-scattering spectroscopy; NIR = near-infrared; FL = fluorescence; OCT = optical coherence tomography.

Pioneering optical studies to characterize breast tumor margins was carried out by Bigio et al [30] where they used reflectance spectroscopy in the UV-Visible range to look at sites within the tumor bed in 24 patients (13 cancer and 59 normal sites). This work was important in that it represented initial evidence of absorption and/or scattering contrast in residual breast cancer. Keller et al published on diffuse reflectance and fluorescence spectroscopy to detect cancerous sites on excised breast tumor margins in 32 patients (145 normal and 34 individual tumor sites), and reported a sensitivity and specificity of 85% and 96%, respectively, for classifying individual sites (not margins) [31]. Haka et al published on Raman spectroscopy of tumor sites on freshly sliced lumpectomy specimens in 21 patients (123 benign and 6 malignant tissue sites) and exploited fat and collagen contrast to achieve sensitivity and specificity of 83% and 93%, respectively for classifying individual sites [32]. Nguyen et al [33] demonstrated that optical coherence tomography detects *ex vivo* margin positivity in 20 patients (11 positive/close margins and 9 negative margins), with sensitivity and specificity of 100% and 82%, respectively by exploiting scattering associated with increased cell density. Nachabe et al [34] used diffuse reflectance spectroscopy to acquire spectra from 102 *ex vivo* samples that consisted of adipose, glandular, fibroadenoma, invasive carcinoma, and DCIS. Using a K-nearest neighbor algorithm, malignant and non-malignant samples were separated with a sensitivity of 94±4% and a specificity of 98±2%.

**Figure 1:**
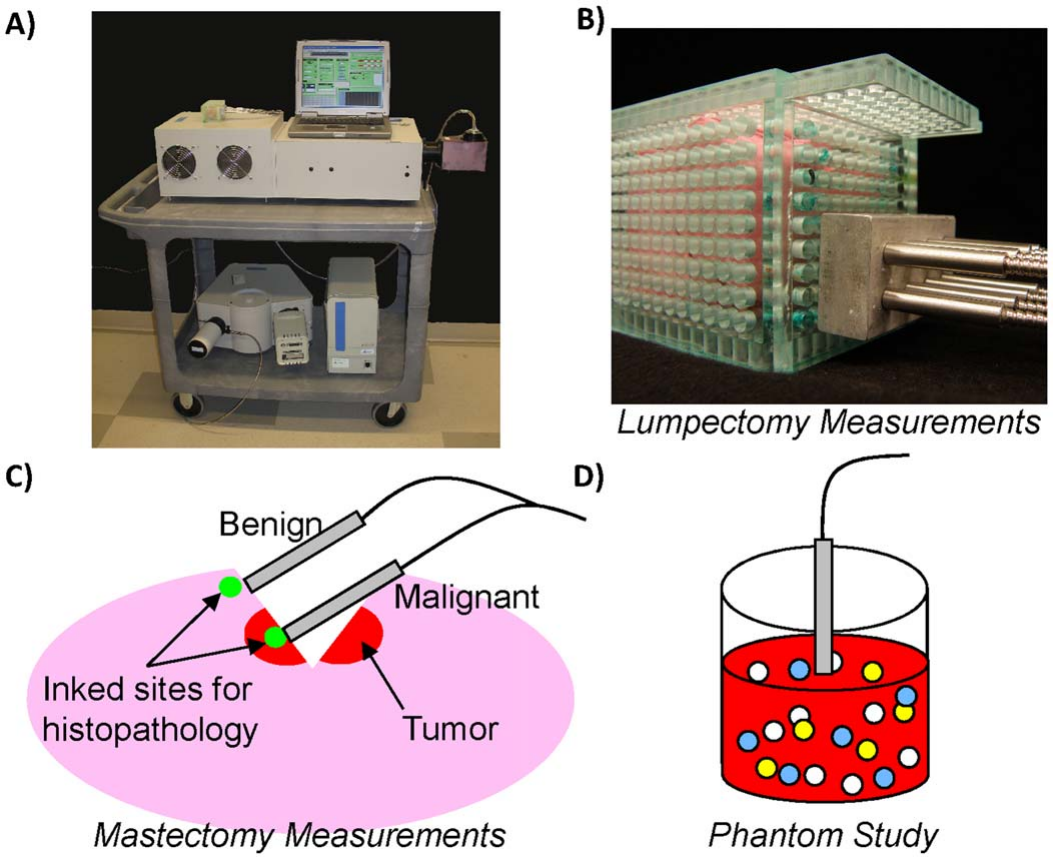
Instrumentation and measurement procedure. A) Photo of the spectral imaging device connected to a laptop. B) Photo of the 8-channel fiber optic probe secured in an aluminum adaptor. For lumpectomy measurements, the probe was interfaced to the tissue surface via holes in the plexi-glass box. C) Cartoon of a mastectomy specimen sliced open to reveal tumor (cross-sectional view). One channel of the probe was placed on grossly benign tissue and another on grossly malignant tissue. The two sites were inked for histopathology after measurements were taken. D) For the patent blue dye phantom studies, one channel of the probe was placed in the center of the vial containing the liquid phantom.

We published recently on using a quantitative diffuse reflectance spectral imaging technique to non-destructively image lumpectomy margins surrounding a mass in 48 patients [35], [36]. What is unique about our published work on breast tumor margin assessment is that we demonstrated the capability to image an entire tumor margin, which has yet to be demonstrated by previously published optical techniques. The engine of this bench-top spectral imaging system is a broadband source that emits at visible wavelengths, an imaging spectrograph, and a CCD camera which are shown in Figure 1A [37], [38]. Light is relayed between the instrument and each discrete site on the margin within a specimen box via an imaging probe (Figure 1B) [36]. The diffuse reflectance spectra *per* site were analyzed with a feature extraction algorithm based on a fast, scalable Monte Carlo model developed by our group [39], [40] to quantitatively determine absorption (β-carotene and hemoglobin) and scattering contrast in the breast. These sources of contrast were used to create tissue morphology maps which were used in a decision-tree model to differentiate positive from negative margins. We reported sensitivity and specificity of 79% and 67% respectively on 55 margins from 48 patients [35], [36] imaged 16±5 minutes post-excision. We have since accrued images from 88 margins in 70 patients and the results are consistent with those reported previously. In summary, optical imaging technologies can aid the surgeon in finding positive margins and they can also be used to guide pathological assessment of tissue and provide insight into where to sample the tissue, thereby improving sampling yield, particularly in larger tumor specimens in both the intra-operative and post-operative setting.

Before this technology can be used in an intra-operative setting or in a post-operative setting, systematic studies have to be performed to determine which surgical and post-surgical factors affect the precision and accuracy with which this technology maps optical contrast. This is true not only for our technology but other technologies, both optical and non-optical that are intended for this application. Specifically, if the technology is to be used on the excised margin (which is the way in which intra-operative pathology is performed), then there must be an understanding of how the presence of the blue sentinel lymph node mapping dye (referred to as patent blue dye) and cautery could influence the primary sources of contrast in the breast. Another important variable to characterize is the impact of the time delay after excision on the primary sources of optical contrast in the breast. Given that all of the recent studies reporting on optical technologies have been carried out on resected tumor margins [32], [33], [35], [41], [42] and the fact that frozen section and post-operative pathology are necessarily carried out on resected specimens, characterizing the effects of these potential sources of error will be important in the context of developing optically based margin imaging tools for use by surgeons and pathologists.

## Materials and Methods

### Ethics Statement and Clinical Protocol

Patients undergoing partial mastectomy were utilized in this study. The study was approved by the Duke University Institutional Review Board (protocol #00017428) and written consent was obtained for every patient. Patient enrollment was limited to patients who had not received a prior surgical excisional biopsy for cancer diagnosis or prior chemotherapy or endocrine therapy. Standard surgical protocol was followed. Following excision, orientation by the surgeon, and specimen mammography, the specimen was placed in a plexi-glass box. Diffuse reflectance spectra were obtained from 8 discrete sites on the tumor margins and all of these sites were inked for histopathology.

Patients undergoing mastectomy were also utilized in this study. The study was also approved by the Duke University Institutional Review Board under the same protocol (protocol #00017428) and written consent was obtained for every patient. Patient enrollment was limited to patients with palpable tumors (approximately >1 cm) who had not received a prior surgical excisional biopsy for cancer diagnosis or prior chemotherapy or endocrine therapy. Standard surgical protocol was followed. Immediately following excision, the breast was inked and the dimensions (anterior-posterior, inferior-superior, and medial-lateral) were measured for standard surgical pathology. A single incision was then made through the posterior or anterior aspect of the mastectomy specimen into the center of the tumor by the surgeon or a board-certified pathologist (JG) present in the operating room. Diffuse reflectance spectra were measured from two locations from each mastectomy specimen; one corresponding to grossly benign tissue and the other grossly malignant tissue as identified by the pathologist. Once measurements were completed, the two sites were inked for histopathology.

The lumpectomies were used to quantify the degree to which each optical endpoint changed over time. Benign sites from both lumpectomies and mastectomies were used to evaluate the effect of cautery (incised mastectomies did not undergo cautery within the measurement area). Finally, because the yield of positive sites was low in the lumpectomy specimens, only mastectomies were used to compare the kinetics in the primary sources of optical contrast between benign and malignant breast tissue.

### Instrumentation

The optical imaging device (Figure 1A–B), previously described in detail [35], [43]–[45], consisted of a 450 Watt Xenon lamp coupled to a monochromator (Jobin Yvon Horiba), a multi-channel fiber-optic imaging probe (designed in-house and custom built by RoMack Inc.), a spectrograph (Jobin Yvon Horiba), and a 2D CCD camera (Jobin Yvon Horiba). There were 8 channels on the multi-channel probe. Each channel had a core of 19, 200 µm (NA = 0.22) illumination fibers surrounded by 4, 200 µm (NA = 0.22) collection fibers with source-detector separations spanning 0.23–1.10 mm. The sensing depth of the probe was previously simulated to be 0.5–1.5 mm for malignant tissue, 0.7–2.2 mm for adipose tissue, and 0.6–1.5 mm for fibro-glandular tissue in the *ex vivo* breast [44]. The device was used in both the mastectomy and lumpectomy studies.

### Optical Measurement of Lumpectomies and Mastectomies

In the lumpectomy study, the specimen was placed in a plexi-glass box and interfaced with the 8 channels of the probe via the holes in the box (Figure 1B). For this study, a diffuse reflectance spectrum (450–600 nm) was collected periodically, with intervals of ≤1 minute (average of 0.93 minutes apart) between each successive spectrum. The first measurement was taken between 2 and 12 (7±3) minutes post-excision. Spectra were measured for as long as possible without interfering with the surgical team (in practice, this was a range of 10–21 minutes).

Figure 1C demonstrates the procedure for making measurements on the mastectomy specimens. After the mastectomy specimen was sliced to expose the tumor, one channel of the 8-channel fiber optic probe was placed on the tumor while another was placed on grossly benign tissue within the excision; the probes were held securely in place with adjustable laboratory clamps. Note that the measurements were made on non-cauterized freshly cut tissue surfaces. For this study, a diffuse reflectance spectrum (450–600 nm) was collected periodically, with intervals of ≤1 minute (average of 0.42 minutes apart) between each successive spectrum. The first measurement was taken between 10 and 27 (17±4) minutes post-excision. Spectra were measured for as long as possible without interfering with the surgical team (in practice, this was a range of 10–32 minutes).

The measurement times were consistent with our previous lumpectomy study [35], [45] where measurements commenced 16±5 minutes after excision. In that study, the total time from excision to the end of margin imaging was 29±15 minutes (average and standard deviation). Each diffuse reflectance spectrum was divided by the CCD integration time, and corrected for daily variations in optical throughput by dividing the tissue spectrum by a spectrum collected from a 99% Spectralon reflectance standard (LabSphere) at each wavelength.

All data were analyzed over a wavelength range of 450–600 nm. Our inverse Monte Carlo model [39], [44], [46] was used to extract the wavelength-dependent optical property spectra (µ_a_ – absorption coefficient and µ_s_’ – reduced scattering coefficient) of each tissue site from the calibrated diffuse reflectance spectrum. Concentrations (denoted with [ ]) of the various breast absorbers were calculated from µ_a_ using Beer’s Law; these included: [oxy-hemoglobin], [deoxy-hemoglobin], hemoglobin saturation (HbSat), total hemoglobin ([THb]), [β-carotene], and the sentinel lymph node mapping dye Lymphazurin™ (referred to throughout as [patent blue dye]). Each reduced scattering coefficient spectrum was further reduced to a scalar value by averaging over the wavelengths from 450–600 nm; this is denoted as <µ_s_′> throughout the manuscript. Additionally, ratios of these tissue parameters were also calculated as endpoints, such as [THb]/<µ_s_′> or [β-carotene]/<µ_s_′>.

### Histopathology

Upon completion of the measurements, the measured sites were inked for histological correlation. The specimens were then transferred to the surgical pathology laboratory for routine pathologic processing, and following routine diagnostic workup the inked sites were evaluated microscopically by the study pathologist (JG). The benign sites were classified as fat, fibro-adipose, fibro-glandular, or mixed/other; mixed/other refers to any site with some combination of fat, collagen, glands, or vessels. The malignant sites were classified as invasive ductal carcinoma (IDC), ductal carcinoma *in situ* (DCIS), or mixed/other; for these, mixed/other refers to sites with some combination of IDC, DCIS, or lobular carcinoma. If tumor cells extended to the inked surface, the margin was considered positive. If they were within 2 mm of the inked surface, the margin was considered close.

### Sample Sizes

From March 2011 to September 2011, lumpectomies were analyzed from 10 patients resulting in 80 sites. A total of 7 sites were excluded due to poor probe-tissue contact. The tissue was submitted for histopathology on the remaining 73 sites. However, histopathology could only be obtained for 61 of the sites. From May 2009 to October 2010, mastectomies from 19 patients were analyzed, resulting in 38 individually-measured tissue sites. The optical parameters were plotted versus time for every site and inspected for trends; 4 sites were removed due to poor probe-tissue contact and/or motion artifacts observed in the data, 2 additional sites (1 patient) were removed because the optical measurements were made 85 minutes after excision which was much longer than the other sites. Of the remaining 32 sites, 20 had microscopic histological confirmation. Samples with histology confirmation were given an overall diagnosis of benign or malignant, and were then given a further classification by specific histological subtype. The breakdown of sample sizes and tissue subtypes is shown in Table 2.

**Table 2:** Sample sizes of histologically-confirmed sites.

	Lumpectomies	Mastectomies
FG	1	1
FA	2	2
Adipose	29	4
Mixed/Other	27	6
*Total Benign*	*59*	*13*
IDC	1	5
DCIS	1	0
Mixed/Other	0	2
*Total Malignant*	*2*	*7*
**Total # of Sites**	**61**	**20**
**Total # of Patients**	**10**	**12**

FG = fibroglandular; FA = fibroadipose; IDC = invasive ductal carcinoma; DCIS = ductal carcinoma in situ.

### Statistical Analysis of Lumpectomies and Mastectomies

The extracted tissue parameters were fit to a longitudinal mixed-effects model, which is an appropriate method for evaluating the trends over time in optical measurements across different tissue types. Longitudinal models were performed in R version 2.7.2 (www.r-project.org) using the lme4 package. The fixed-effect terms in the models were the time from surgical excision of the specimen and the histological subtype of the measured site. This model resulted in a fitted slope for every measured site. In all tests of main effects and interactions, statistical significance was considered to be p<0.05.

#### Lumpectomy kinetics

Our first step was to characterize how the optical properties changed over time in lumpectomies. To calculate the rate of change, sample-specific slopes were estimated from the mixed model along with their significance. To determine the most robust optical parameters for imaging lumpectomy margins, the percent change was calculated for all lumpectomy sites over a 30 minute time window (to be consistent with the average time it took to fully image the lumpectomy margins in our previous study). The percent change was used for this comparison since rates of change cannot be compared across tissue parameters since they have different units and magnitudes. The percent change was calculated by dividing the rate of change by the absolute value of the intercept from the fitted data. The association between the first measurement and time from excision were evaluated using Spearman correlations.

#### Cautery

Cautery artifacts on tissue in H&E stained histology slides have proved to be problematic for diagnosing surgical margin status [47]. Given that cautery is visible under H&E we questioned how this might impact optical imaging of tumor margins. To determine the effect of cautery on the optical parameters, the initial values and the rates of change of the benign sites were compared between lumpectomies and mastectomies using Wilcoxon rank-sum tests. Lumpectomies and mastectomies were compared since measurements were made from the cauterized lumpectomy surfaces versus the non-cauterized incised mastectomy tissue.

#### Effects of tissue histology on kinetics

In terms of margin assessment, it is important to ensure that contrast between benign and malignant regions is preserved over time and that the rate of change is not different between tissue types. Ideally this analysis would have been carried out with lumpectomy specimens; however, isolating malignant sites on a lumpectomy margin is challenging. Therefore, to compare the rates of change between benign and malignant tissue, we utilized mastectomy specimens that could be incised to reveal gross tumor. Likelihood ratio tests were used to determine whether the rate of change was dependent on tissue type; specifically whether there were differences in the rates of change in the optical parameters between benign and malignant tissue. Spearman correlations were computed for both benign and malignant sites between the first measurement and time from excision.

### Patent Blue Dye Simulations and Phantom Studies

**Figure 2:**
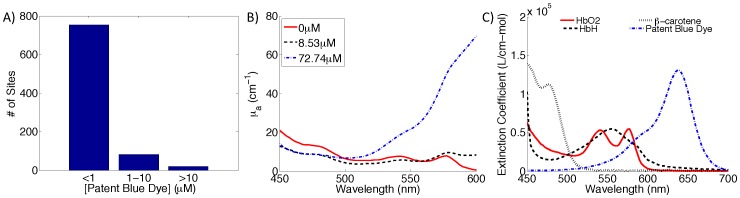
Characterization of [patent blue dye]. A) Histogram of the extracted [patent blue dye] for the site-level data (854 sites). Maximum extracted [patent blue dye] was 72.7 µM. B) Extracted absorption coefficient spectra for 3 different fat sites with varying [patent blue dye]. C) Extinction coefficients for oxy-hemoglobin (HbO2), deoxy-hemoglobin (HbH), and β-carotene measured by Prahl [54]; and patent blue dye.

Patent blue dye is used for sentinel lymph node mapping with an extinction coefficient that partially overlaps with the alpha and beta bands of oxygenated and deoxygenated hemoglobin (Figure 2C). Although the inverse Monte Carlo model [39], [40] accounts for patent blue dye by assuming this to be a primary absorber in breast tissue, it is unknown how accurately the model can extract [THb], [β-carotene], and scattering in the presence of varying concentrations of patent blue dye. A forward Monte Carlo model was used to obtain simulated diffuse reflectance spectra with known absorption and scattering levels. From our previous site-level study [43] with 854 sites from lumpectomy specimens, the median, 25th and 75th quantiles were computed for <µ_s_’>, [THb], and [β-carotene]. These 3 levels were [4.85, 6.68, 9.15 cm^−1^] (<µ_s_’>), [16.97, 31.03, 55.09 µM] (THb), and [10.29, 16.29, 24.37 µM] (β-carotene). The full range of patent blue dye concentrations were also determined from the site-level data and are shown in Figure 2A. The majority of the measured sites have less than 5 µM of patent blue dye but go as high as 72.7 µM. The effects of these different concentrations are reflected in the absorption spectra of Figure 2B where µ_a_ at 600 nm increases with increasing [patent blue dye]. Therefore, for the simulations, a diffuse reflectance spectrum was created for every combination of absorption and scattering levels, and [patent blue dye] was input in 10 µM increments from 0 to 70 µM. This resulted in 216 simulated spectra (3×3×3×8).

The simulated spectra were then inverted in the same manner as the clinical data to extract <µ_s_’> and the concentrations of THb, β-carotene, and patent blue dye. This inversion process used known extinction coefficients for β-carotene, oxy-hemoglobin, and deoxy-hemoglobin. The extinction coefficient for patent blue dye was measured by our group previously. To determine the accuracy of the model at extracting the absorbers and scattering information with varying degrees of [patent blue dye], the percent error was calculated between the expected data and extracted simulated data.

We have previously tested the accuracy of our system using phantoms containing hemoglobin, crocin (a substitute for β-carotene), and polystyrene spheres (a scatterer), and showed that all parameters could be extracted with <15% error [44]. Similarly, tissue mimicking phantoms consisting of 1.025 µm diameter polystyrene spheres (Polysciences), hemoglobin (H0267– Sigma-Aldrich), crocin (17304 Standard Fluka, Sigma-Aldrich), and Lymphazurin™ (TycoHealthcare) were created to access the effect of [patent blue dye] on the accuracy of the Monte Carlo model [39], [40] to extract [THb], [β-carotene], and <µ_s_’>. A single channel of the probe was used in these studies, as shown in Figure 1D, since all channels have similar illumination and collection geometries. The absorber concentrations and scattering for the phantoms were also based on the ranges seen in the site-level clinical study [43], [44]. However, for the phantom study the median scattering level, and minimum [THb] and [β-carotene] levels were chosen. The lowest levels were selected because, theoretically, these would be most affected by high concentrations of patent blue dye. The actual absorber and scattering levels for the phantom were as follows: 5.81 cm^−1^ (<µ_s_’>), 16.69 µM ([THb]), 11.23 µM ([β-carotene]), and 0–79 µM ([patent blue dye]). One phantom containing the spheres, hemoglobin, and crocin was made; and 12 titrations with increasing [patent blue dye] were added. The inverse Monte Carlo model was used to extract the concentrations of the absorbers and <µ_s_’>, and the percent error was calculated between the expected and extracted values to determine the effect of varying concentrations of patent blue dye..

## Results

### Optical Parameters Affected by Excision in Lumpectomies

**Figure 3:**
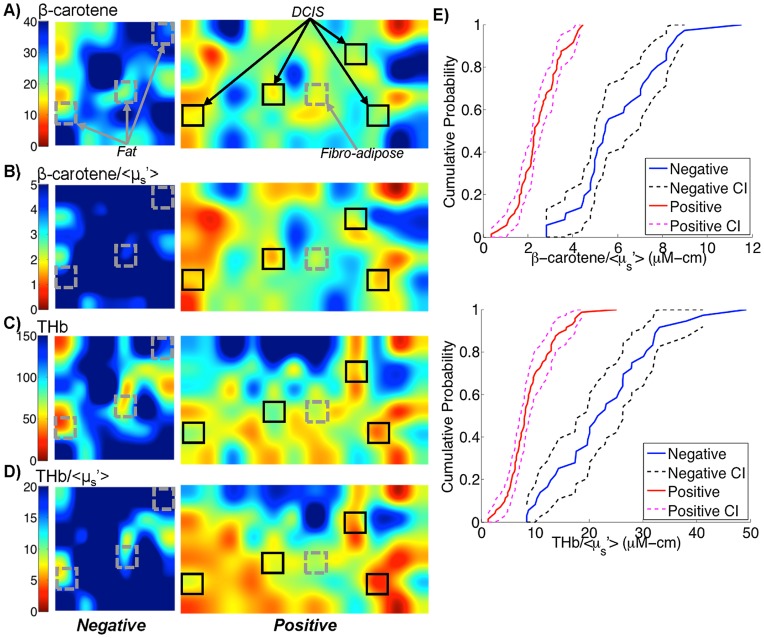
Example data acquired from lumpectomy margins in our previous study [**35**] **.** A–D) 50× bicubic interpolated images of β-carotene, β-carotene/<µ_s_’>, THb, and THb/<µ_s_’> from a negative margin (3.5 cm×3.5 cm) and a positive margin (3.5 cm×6.5 cm). Benign (fat and fibro-adipose tissue) and malignant (ductal carcinoma in situ – DCIS) sites are highlighted. E) Cumulative distribution functions of the pixels in the negative and positive margins from the representative images along with their confidence intervals (CI).

The purpose of doing these studies was to identify the optical parameters that are least affected by kinetics, the presence of patent blue dye, and cautery for optical margin assessment of lumpectomy specimens. Figure 3A–D show representative optical images of [β-carotene], [THb], and the ratios of [β-carotene] to <µ_s_’> and THb to <µ_s_’> for a negative (no residual carcinoma within 2 mm of the surface) margin and a positive margin. Histologically confirmed sites are highlighted corresponding to adipose, fibroadipose, or ductal carcinoma *in situ* (DCIS). Empirical cumulative distribution functions are shown in Figure 3E representing the distribution of the data in the negative and positive representative images. Both the margin-level data and site-level data show that [β-carotene]/<µ_s_’> and [THb]/<µ_s_’> decrease with malignancy and these were important parameters in differentiating margins in our previously published 48-patient study [35].

Our first set of analyses examined the kinetics of lumpectomy specimens to determine how optical images of margins are impacted. The linear longitudinal model was used to fit lumpectomy data from all 10 patients (Table 3). Visually, β-carotene, <µ_s_’>, THb, and the ratios showed little change over time. HbSat as well as, oxy-hemoglobin and deoxy-hemoglobin exhibited marked changes over time and were not linear throughout the entire measurement window (data not shown). Therefore, oxy and deoxy-hemoglobin, and HbSat are not shown for the remainder of this manuscript for any analyses.

**Table 3:** Rates of change in lumpectomy and mastectomy sites.

	Lumpectomy	Mastectomy		
	Benign	Benign	Malignant	p-value
β-carotene (µM/min)	−0.027±0.143	0.015±0.062	0.002±0.045	0.627
<µ_s_’> (cm^−1^/min)	^−^0.034±0.040	^−^0.010±0.019	^−^0.008±0.023	0.829
β-carotene/<µ_s_’> (µM-cm/min)	0.010±0.020	0.005±0.009	0.001±0.005	0.304
THb (µM/min)	−0.537±0.750	−0.036±0.191	−0.123±0.112	0.256
THb/<µ_s_’> (µM-cm/min)	−0.062±0.098	−0.005±0.043	−0.011±0.012	0.698

THb = total hemoglobin; <µ_s_’> = reduced scattering coefficient.

Rate of change per minute (fitted values from the model) for each tissue parameter of the benign (n = 13) and malignant (n = 7) sites measured in mastectomy specimens and benign (n = 59) sites measured in the lumpectomy specimens. Reported values indicate the average ± standard deviation. P-values indicate the statistical differences in the rate of change between the benign and malignant mastectomy sites.

**Figure 4:**
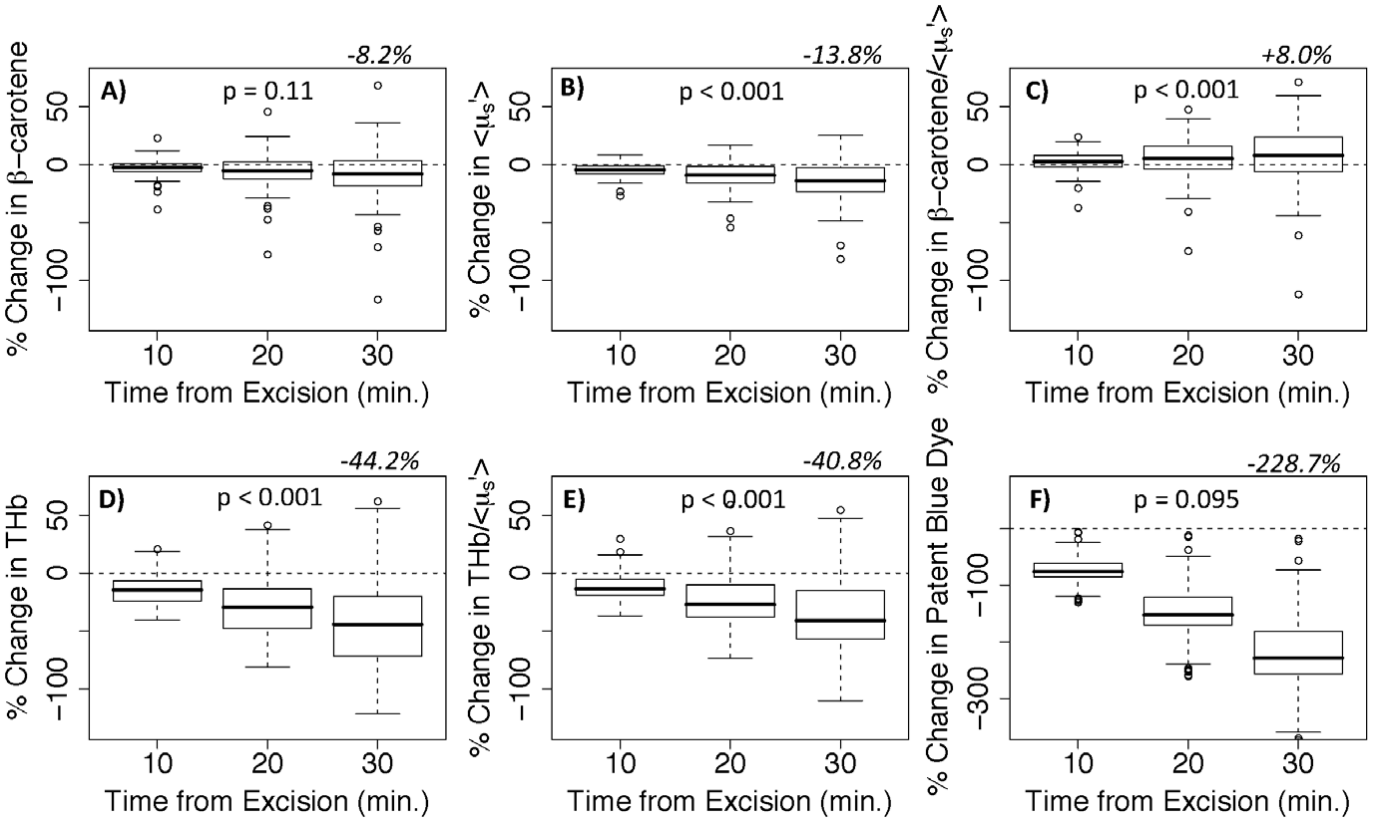
Percent change in each tissue parameter versus time from excision. Predicted values of percent change; calculated as the fitted rate of change divided by the absolute value of the fitted intercept, multiplied by time from excision. Data is from histologically-confirmed lumpectomy sites (not all outliers are shown). The median percent change at 30 minutes is noted for each parameter along with a p-value denoting the significance of the fitted slope coefficient.

The rate of change could not be compared across tissue parameters because the units and magnitudes of the variables were not the same. Therefore, the percent change at various time points post-excision was calculated to identify the optical parameters with the smallest percent change. Figure 4 shows the percent change in the optical parameters. A maximum of 30 minutes is shown here to correspond with our previous lumpectomy study [35], [43]–[45] where the average amount of time elapsed between excision and the end of imaging was 29 minutes. These results show that [β-carotene], <µ_s_’>, and [β-carotene]/<µ_s_’> had the lowest percent changes over a 30 minute time window (median percent changes of −8.2, −13.8, and −8.0%). [THb] and [THb]/<µ_s_’> had larger percent changes of −44.2% and −40.8% respectively; and [patent blue dye] had the largest percent change of −228.7%. This data was also evaluated for correlations between the first optical measurement and the time from excision. Spearman correlations were computed for all measured sites and no significant correlation was found between any of the optical parameters and time from excision to measurement, except for [patent blue dye] with p = 0.0007. This was likely due to patent blue dye draining from the tissue after excision.

### Effects of Patent Blue Dye on Optical Absorption and Scattering in the Breast

**Figure 5:**
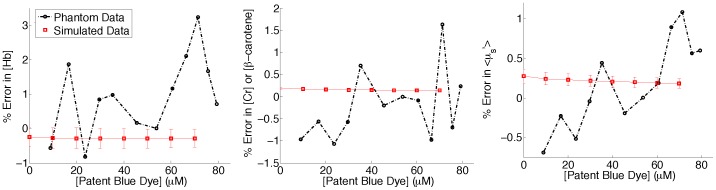
Average percent errors for Monte Carlo simulated data and phantom data. Data is shown for a single reference “phantom” (<µ_a_> = 3.85 cm^−1^, <µ_s_’> = 6.79 cm^−1^ for the simulated data; <µ_a_> = 3.02 cm^−1^, <µ_s_’> = 5.81 cm^−1^ for the phantom data). Simulated: 216 diffuse reflectance spectra were created consisting of 3 levels of scattering (4.85, 6.68, 9.15 cm^−1^), THb (16.97, 31.03, 55.09 µM), and β-carotene (10.29, 16.29, 24.37 µM) and 8 levels of patent blue dye (0∶10:70 µM). Phantom: patent blue dye was titrated 12 times (0–79 µM) into a phantom consisting of 5.81 cm^−1^ (<µ_s_’>), 16.69 µM (THb), and 11.23 µM (β-carotene).

In Figure 4 we showed that [patent blue dye] had the highest percent change over a 30 minute period and that it was correlated with time from excision. This led us to question whether the amount of patent blue dye would impact the other optical absorbers and scattering. Therefore, Monte Carlo simulations and a tissue mimicking phantom study were carried out to address this question. The simulated data covered the full range of absorption and scattering levels seen in our previous breast studies [43], [44], while the phantom data was for a subset of the breast optical properties that would result in the worst-case scenario, i.e. where patent blue dye dominates the absorption spectrum. The percent error in [THb], [β-carotene] (crocin for the phantoms), and <µ_s_’> as a function of [patent blue dye] is shown in Figure 5 for both simulated and phantom data. The simulated data had negligible error, while the phantoms had slightly higher error attributed to experimental measurements. The simulated and phantom data both showed <3.3% error in extracted [THb], [β-carotene], and <µ_s_’> even in the presence of high concentrations of patent blue dye (up to 80 µM). With [THb] and [β-carotene] there did not appear to be any relationship of error with increasing [patent blue dye]. In the phantom data, when [patent blue dye] was approximately 10 µM, <µ_s_’> was underestimated by the model and as [patent blue dye] was increased, <µ_s_’> was overestimated. This should not be a concern though as the percent error was <1.5% and the simulated results showed no trend. Overall, these results indicate that patent blue dye in concentrations up to 80 µM do not impact the ability to quantify [THb] or [β-carotene], or <µ_s_’> within the wavelength range of 450–600 nm.

### Effects of Cauterization on the Optical Parameters

**Figure 6:**
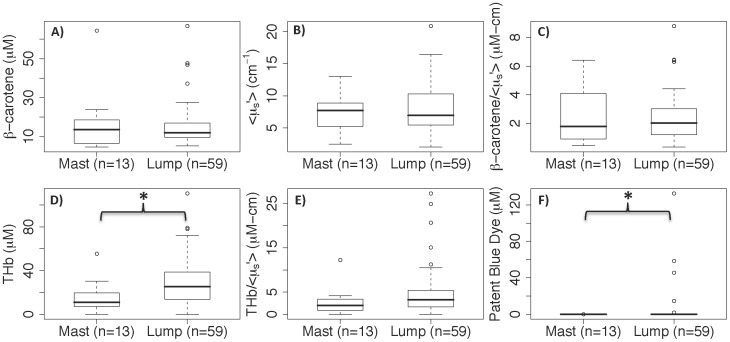
Optical parameters of the first measured time point. Optical parameters of the first time point from the histologically-confirmed benign sites of mastectomies (Mast) and lumpectomies (Lump). * indicates p<0.05.

**Figure 7:**
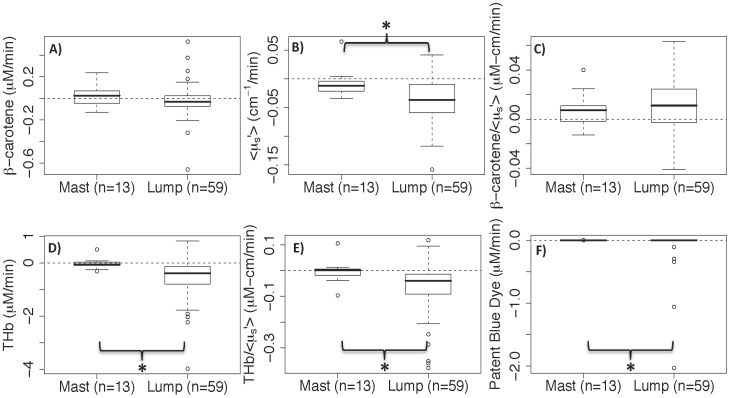
Rate of change in the tissue parameters. Rate of change (fitted values from the model) in the tissue parameters from the histologically-confirmed benign sites of mastectomies (Mast) and lumpectomies (Lump) constrained to a time window of 10 min for all sites. * indicates p<0.05.

Figure 6 shows the initial measurement of each optical endpoint separated by specimen type (lumpectomy or mastectomy). [THb] and [patent blue dye] were the only parameters that were significantly higher (p = 0.013 and 0.0004, respectively) in the lumpectomies compared to mastectomies. We also examined the differences in the rates of change (constrained to a 10 minute time window) between mastectomy and lumpectomy benign sites (Figure 7). [β-carotene] and [β-carotene]/<µ_s_′> were the only parameters that were not significantly (p = 0.13 and 0.36 respectively) different between the two types of specimens.

**Figure 8:**
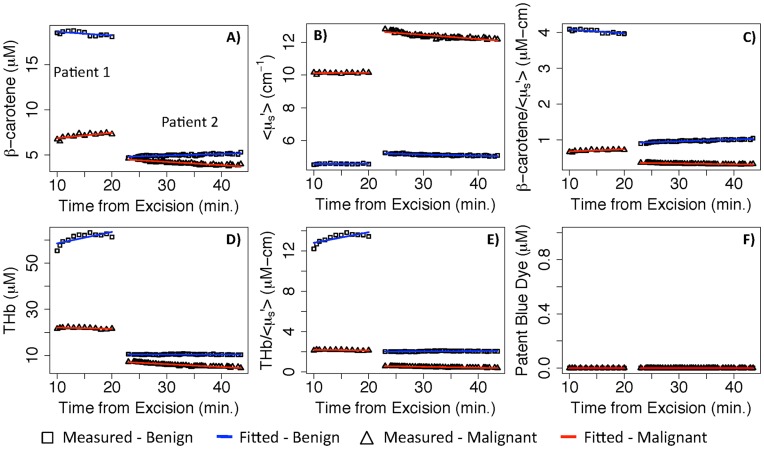
Example plots of kinetics in benign and malignant tissue. Example plots of the tissue parameters versus time for four histologically known sites from two mastectomy patients. Symbols indicate the measured data lines are the model fits for the benign and malignant tissues.

**Figure 9:**
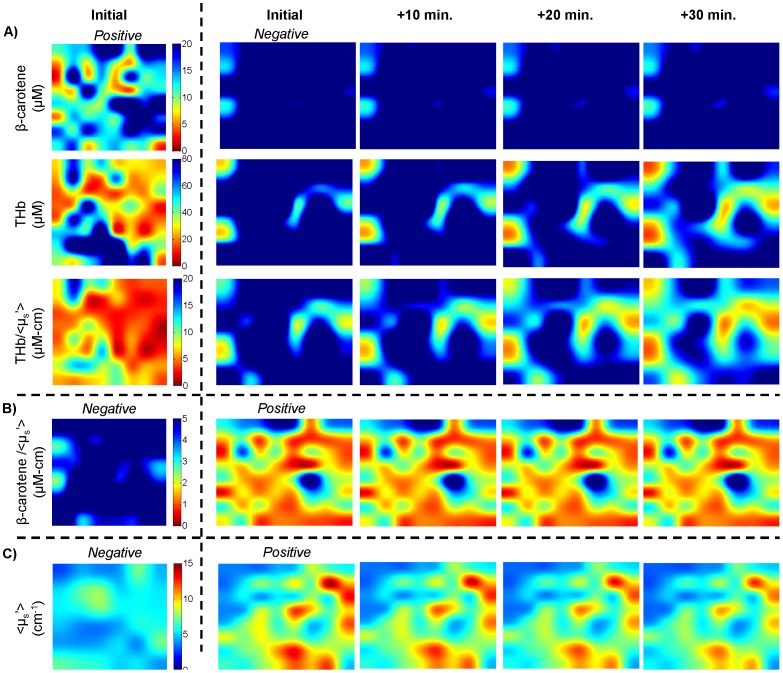
Effects of time on *ex vivo* spectral imaging. 50x bicubic interpolated images of a negative and positive margin from different patients, where the “initial” images of the margins were imaged at approximately the same time points post-excision. The “initial” images represent the actual data measured. The median percent change at 10, 20, and 30 minutes was applied to either the negative or positive image to show how an image would change if measured at various time points beyond the “initial” image time point. A) For β-carotene, THb, and THb/<µ_s_’>, the negative margins have higher values and the kinetics decrease over time. Therefore, the percent change is applied to the negative margin to show decreasing contrast (worst case scenario). B) For β-carotene/<µ_s_’> the negative margins have higher values and the kinetics increase over time. C) For <µ_s_’> the positive margins have higher values but the kinetics decrease over time.

**Table 4:** Comparison of the percent change over time versus the percent difference in benign and malignant tissue.

	% Change			%Difference		
	At 10 min.	At 20 min.	At 30 min.	A vs. P	FA vs. P	FG vs. P
β-carotene (µM)	−2.7	−5.5	−8.2	29.8	22.7	9.7
<µ_s_’> (cm^−1^)	−4.6	−9.2	−13.8	−27.8	−9.7	29.8
β-carotene/<µ_s_’> (µM-cm)	2.7	5.3	8.0	14.1	−7.3	−88.3
THb (µM)	−14.7	−29.4	−44.2	−101.2	−105.5	−92.3
THb/<µ_s_’> (µM-cm)	−13.6	−27.2	−40.8	−92.2	−99.5	−108.3

THb = total hemoglobin; <µ_s_’> = reduced scattering coefficient; A = adipose; P = positive malignant; FA = fibroadipose; FG = fibroglandular.

Comparison of the percent change in each optical parameter at 10, 20, and 30 minutes post-excision to the percent differences between 1) adipose and positive sites, 2) fibroadipose and positive sites, and 3) fibroglandular and positive sites from our initial site-level study [43], [44]. A negative value in the percent difference indicates that positive sites were greater; a positive value means the benign tissue was greater.

### Kinetics in Benign and Malignant Tissue

Figure 8 shows representative plots of two sites from two different patients, one measured with the shortest time from excision and the other with the longest time from excision. For each patient the histologically-confirmed benign and malignant site are shown. From the measured data we see that [β-carotene], <µ_s_′>, and [β-carotene]/<µ_s_′> were relatively invariant with time as was observed above. [THb] and [THb]/<µ_s_′> were also relatively invariant, although the benign site of Patient 1 had a slightly higher slope. The fitted data shown in this figure were from the longitudinal model. The model provided excellent fits to [THb], [β-carotene], <µ_s_′>.

To quantify how much the tissue parameters changed over time, the data from the mastectomy sites was fit with the longitudinal model. Table 3 shows the fitted rate of change in the tissue parameters; a negative value indicates a parameter that decreased over time and a positive value indicates a parameter that increased with time. Most of the tissue parameters decreased with time and the rate of change was similar between the benign and malignant mastectomy sites. An interaction test was used to determine if the histology of the measured sites affected the rate of change. The results indicate that the rate of change did not differ significantly between the benign and malignant sites for any of the tissue parameters. This data was also evaluated for correlations between the first optical measurement and the time from excision. Spearman correlations were computed for both benign and malignant sites and no significant correlation was found between any of the optical parameters and time from excision (lumpectomy or mastectomy).

### Impact of Kinetics on Optical Contrast for Margin Assessment

The results in this manuscript indicate that [β-carotene] and [β-carotene]/<µ_s_’> are the most robust variables but still change ∼8% in 30 minutes. The question that arises is: what happens to contrast between negative and positive margins if a negative margin is imaged immediately after excision and a positive margin is imaged 30 minutes after excision, or vice versa? Figure 9 helps to illustrate the extent to which kinetics affect optical contrast. Images of a positive and negative margin from two different lumpectomies imaged at approximately the same time points post-excision are shown. The “initial” image is the actual parameter map that was measured. The median percent change for each variable at 10, 20, and 30 minutes post-excision (data from Section 3.2) was applied to either the negative or positive image to artificially decrease contrast. In Figure 9A this was implemented by multiplying the negative image by the median percent change. For [β-carotene], [THb], and [THb]/<µ_s_’>, the percent change was in the negative direction and positive margins have lower values for these variables. To show the worst-case scenario, the percent change was applied to the negative margin to decrease contrast. In Figure 9B, [β-carotene]/<µ_s_’> is lower in positive margins but increases over time; therefore, the percent change was applied to the positive margin to decrease contrast. In Figure 9C, <µ_s_’> is higher in positive margins but decreases over time, so the percent change was applied to the positive margin. These images show that an 8% change in [β-carotene] and [β-carotene]/<µ_s_’> does not alter the contrast between the negative and positive margin. <µ_s_’> contrast also does not change significantly; however the initial contrast is not as apparent. By 30 minutes, the >40% change in [THb] and [THb]/<µ_s_’> greatly reduces the differences between the positive and negative margin; however, contrast is still preserved.

Using our previous site-level data [43], [44], a percent difference was calculated between adipose and positive malignant sites; fibroadipose and positive sites; and fibroglandular and positive sites. These values were compared to the percent change at 10, 20, and 30 minutes for the lumpectomy kinetics data (Table 4). The percent change is smaller than the percent difference for all optical parameters, indicating that optical contrast should be preserved within a 20 minute time window.

## Discussion and Conclusions

Quantitative spectral imaging can be used to accurately quantify optical parameters related to tissue morphology. This technology is not only important for breast margin assessment but also for other applications where optical devices are used on excised tissues. A number of other groups have investigated the use of diffuse reflectance spectroscopy in the measurement of *ex vivo* breast tissue [48]–[53]. However, there is a lot of variation in the types of breast specimens (biopsies, lumpectomies, mastectomies, reduction mammoplasties) that have been measured, whether they had been stored in solutions and/or partially processed, and the amount of time that lapsed post-excision before measurements were made. All of these reports involved spectroscopic measurement of breast tissues over a wide range of intervals (<30 minutes to 12 hours) after excision from the body. These changes may be reflected in the optical measurements of the tissue, and the lack of consistency in measurement time intervals post-excision in the reported studies could make it difficult to confidently compare results across studies. The work presented here provides a framework in which investigators using similar technologies can interpret data, design experiments and conduct their own quality control measurements.

In our previous margin-level study [35], [43]–[45], where we performed quantitative diffuse reflectance spectral imaging of *ex vivo* breast lumpectomy margins, tissue kinetics and cautery were not accounted for explicitly in the analysis of those data. It is not surprising we found that the ratios of [β-carotene]/<µ_s_′> and [THb]/<µ_s_′> were the best parameters to differentiate cancer-free margins from margins that contained residual cancer (sensitivity = 79.4% and specificity = 66.7%) [35]. From the current study we determined that HbSat cannot be fit with a linear model due to excessive changes in oxygenated and deoxygenated hemoglobin post-excision. This is likely due to oxygen being consumed by the metabolically active tissue immediately after excision. Although HbSat may be a useful *in vivo* parameter for determining tumor hypoxia, or for examining the local microenvironment, or even for margin assessment of the resected cavity, it is not reliable in *ex vivo* margin assessment of breast tissue specimens. In this study we show that [THb] and [THb]/<µ_s_′> are less likely to be affected by post-excision kinetics than HbSat or [patent blue dye], though both variables had >40% change in a 30 minute time window. Interestingly, the percent differences between positive malignant sites versus adipose, fibroadipose, and fibroglandular sites were much larger than the percent change in 30 minutes. In fact, it would have taken 63 minutes for the percent change in [THb] to exceed the percent difference between positive malignant and fibroglandular sites. Therefore, parameters involving [THb] may have large percent changes over time but the contrast between benign and malignant tissues appears to be greater within a reasonable time window. [β-carotene], <µ_s_′>, and [β-carotene]/<µ_s_′> were least affected by kinetics (<14% in 30 minutes).

The results from both the simulated and phantom data for [patent blue dye] indicate that [patent blue dye] up to 80 µM does not impact the extractions of [THb], [β-carotene], or <µ_s_′> from the diffuse reflectance spectra; again, the highest concentration of patent blue dye seen in the previous lumpectomy study was 72.7 µM. Although the errors were higher in the phantom data (as would be expected), there was no trend in the percent error with increasing [patent blue dye].

In terms of tissue cauterization, we found that initial measurements of [THb] were significantly higher in the benign sites of the cauterized lumpectomies compared to the mastectomies. This initial difference could be due either to varying excisional times for mastectomy and lumpectomy procedures or due to cauterization. Since we observed no significant correlation between the initial value and time from excision, we assume that this difference in [THb] is due to cauterization of the vasculature to prevent blood from draining out of the vessels as rapidly as it would in mastectomy specimens.

For all tissue parameters, the rate of change was not significantly different between the benign and malignant sites. This is an important finding for margin assessment which indicates that optical contrast between benign and malignant regions of a margin will be preserved, regardless of the time when the margin is imaged over a 30 minute window. We also showed that there was no correlation between the time from excision and the initial value (or first measurement) of the optical data. This suggests minimal change in the data within the time window that we examined (17±4 minutes post-excision and measured for 10–32 minutes). Additionally since there was no significant difference between the lumpectomies and mastectomies for [β-carotene] and [β-carotene]/<µ_s_′>, we can extrapolate these findings to benign and malignant tissue in cauterized lumpectomies.
